# Metasurfaces: physics and applications in wireless communications

**DOI:** 10.1093/nsr/nwad164

**Published:** 2023-06-06

**Authors:** Vasileios G Ataloglou, Sajjad Taravati, George V Eleftheriades

**Affiliations:** The Edward S. Rogers Sr. Department of Electrical and Computer Engineering, University of Toronto, Toronto, ON M5S 3G8, Canada; Clarendon Laboratory, Department of Physics, University of Oxford, Oxford OX1 3PU, UK; The Edward S. Rogers Sr. Department of Electrical and Computer Engineering, University of Toronto, Toronto, ON M5S 3G8, Canada

**Keywords:** metasurface, beamsteering, beamforming, wireless communication, time modulation, wave engineering

## Abstract

The ever increasing number of wireless devices and systems has led to a crowded spectrum and increased the demand for versatile and multi-functional wireless apparatuses. Recently, metasurfaces have been explored as a prominent technological solution to the current paradigm of spectrum scarcity by opportunistically sharing the spectrum with various users. In general, metasurfaces are passive/dynamic, ultra-compact, multi-functional and programmable structures that are capable of both reciprocal and nonreciprocal signal-wave transmissions. The controllability and programmability of such metasurfaces are governed through DC bias and occasionally a radio-frequency modulation applied to the active components of the unit cells of the metasurface, e.g. diodes and transistors. This article overviews some of the recently proposed passive and dynamic metasurfaces and shows that metasurfaces can enhance the performance of wireless communication systems thanks to their unique physical features such as real-time signal coding, nonreciprocal-beam radiation, nonreciprocal beamsteering amplification and advanced pattern-coding multiple access communication.

## Teaser

Metasurfaces can enable faster and more reliable wireless communications in the future through their emerging capabilities to effectively manipulate electromagnetic waves in space and time.

## INTRODUCTION

The ever-growing communication traffic is caused by emerging massive-data applications, e.g. autonomous vehicles, tactile internet, virtual reality and augmented reality. However, 5G communication systems are incapable of efficiently supporting such applications. Potential technologies for 6G mobile communication systems include terahertz-band communications, ultra-massive multiple-input multiple-output (MIMO) antennas, block-chain-based spectrum sharing, quantum computing, orbital angular momentum multiplexing, a light-to-microwave transmitter for hybrid wireless communications [1], broadband wireless communication via a space-time-varying polarization-converting metasurface [2], directly wireless communication of human minds via a mind-controlled programming metasurface [3], metasurface-assisted wireless communication with physical-level information encryption [4] and programmable metasurface-based signal sharing [5,6]. Metasurfaces can take many forms, including reconfigurable, programmable, reciprocal/nonreciprocal and signal-boosting engineered surfaces that are capable of steering electromagnetic waves [7,8]. They represent an emerging technology for tailored and advanced coding of electromagnetic waves.

Metasurfaces have recently drawn enormous attention due to their extraordinary capability and potential in wave processing across a wide frequency range, from microwaves to terahertz [9–20]. Such metasurfaces are composed of subwavelength constituents with controllable parameters and adjustable responses (e.g. phase, amplitude, angle of transmission, polarization and operation frequency) through an external bias field in a real-time reconfigurable manner. Over the past few years, an ever increasing research effort has been made on the applications of metasurfaces to wireless communications. Metasurfaces have been employed for the realization of novel and highly efficient wireless transceiver schemes, resulting in a paradigm shift of the transceiver design and reduced hardware cost of wireless communication systems. In particular, microwave metasurface-based wireless communication systems are of great interest. This is because microwave frequencies represent the most favourable frequency ranges for wireless communication, due to the relatively low path loss, non-line-of-sight operation and small transceiver component sizes (e.g. antennas and transmission lines). This study presents a comprehensive list of metasurface-based communication systems that provide an exceptional platform for efficient and programmable data transmission and coding.

Currently, prototypes of reconfigurable and programmable metasurfaces are being demonstrated and startup companies have embarked on developing the fundamental technology that covers a wide range of applications. This paper overviews and analyzes different metasurfaces with distinct features and functionalities that can cover a wide range of requirements of future metasurface-assisted communications. In the following, metasurface-based communication schemes are first described. Next, the paper focuses on metasurface implementations that enable these schemes. Starting from passive and static designs and moving to more complex reconfigurable or time-modulated metasurfaces, we describe the physics, design and possible applications in urban, satellite and cellular wireless communications.

## METASURFACE-BASED COMMUNICATION SCHEMES

Electromagnetic metasurfaces are two-dimensional arrays made of unit cells that are engineered in a way to introduce properties that are not readily available in naturally occurring materials. Reconfigurable metasurfaces are deeply subwavelength in thickness and electrically large in their transverse size, and comprise scattering unit cells. The specific arrangements and bias of the unit cells determine how the metasurface transforms the incident wave into specified but arbitrary reflected and transmitted electromagnetic waves. Such metasurfaces should be capable of providing both reciprocal and nonreciprocal transmission and reflection of signal waves [21], as well as altering the spectrum of the incident wave by generating specified new frequencies. The unit cells of metasurfaces may be loaded by active components such as diodes (e.g. varactor, pin and Schottky diodes) or transistors that may be biased through both direct current (DC) and occasionally augmented by radio-frequency (RF) modulation signal waves. The DC biasing signal is usually employed to change the operation point of the diodes (e.g. average capacitance of varactors), while the RF biasing signal provides a time modulation signal for temporal or spatiotemporal modulation of the effective permittivity or conductivity. Both DC and RF biasing signal waves can then be leveraged for controlling the characteristics and response of the unit cells and the operation, and functionality of the metasurface. Eventually, the real-time full control of the metasurface can be carried out by a computer processor possibly running an artificial intelligence application or a field-programmable gate array.

Transmissive and reflective metasurfaces are placed on top or inside buildings to facilitate an effective communication between different parts of the network, that is, small-cell base stations, macro base stations and satellites. Transmissive metasurfaces and reflective metasurfaces are capable of introducing various functionalities such as active beamsteering, signal-wave amplification, frequency conversion, channel allocation and coding. The reflective metasurface and transmissive metasurface in links may possess distinct characteristics and functionalities such as beamsteering, signal boosting, frequency conversion, real-time signal coding, multiple access communication and nonreciprocal transmission and reflection. The required functionality of each metasurface is governed by the core of the system in a software-controlled and programmable manner.

An efficient and real-time beamsteering guarantees that all the static and mobile elements of the communication system are efficiently covered, whereas signal boosting and amplification enhance the efficiency and quality of the communication. Furthermore, frequency conversion may be required in some parts of the communication network for the sake of bandwidth enhancement or adaptation of the signal wave to the communication channel for lower path loss. Real-time signal coding and multiple access communication are also essential parts of a secure communication network that may be required to guarantee a secure link. Finally, nonreciprocal signal-wave transmission and nonreciprocal-beam radiation are appealing functionalities that can drastically increase the flexibility and capacity of the communication network.

**Figure 1.**
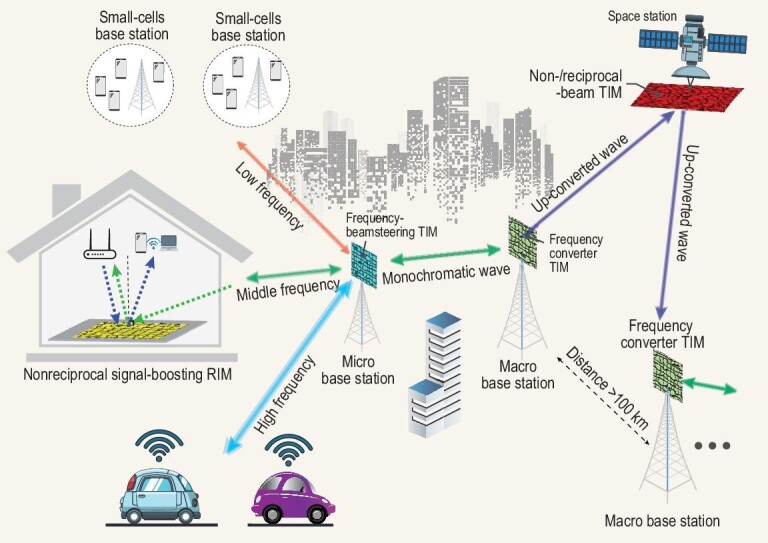
Modern wireless communications in urban areas based on active multi-functional and polychromatic transmissive and reflective metasurfaces. TIM and RIM stand for transmissive and reflective intelligent metasurfaces, respectively. Reproduced from ref. [22].

Figure 1 illustrates a possible scenario of a metasurface-assisted wireless communication in urban areas using active multi-functional metasurfaces [22]. In this scenario, nonreciprocal, frequency-converting and frequency-beamsteering metasurfaces are employed to enhance the versatility and efficiency of the communication network. Metasurfaces are highly versatile components that can play a critical role in meeting the requirements of future wireless communication systems. As shown in Fig. 1, metasurfaces are capable of performing a wide range of functions that are critical for future wireless communication systems. These functions include surface-assisted communication enhancement, [6,9–11], nonreciprocal wave transmission and reflection [12,23], beamsteering [24], signal-wave amplification [13,25,26], intelligent signal management and power handling [14], frequency conversion [15,27–29], analogue and digital signal modulation [18,30], signal processing, and direction of arrival estimation [31]. Metasurfaces offer several advantages over traditional components, such as being multi-functional, highly efficient, intelligent and compact. With their ability to manipulate electromagnetic waves at the subwavelength scale, metasurfaces can enable the development of new wireless communication systems that are faster, more reliable and more efficient than current technologies. For example, metasurfaces can be used to achieve nonreciprocal wave transmission and reflection, which is essential for designing spatial isolators and circulators. These spatial components are critical for preventing interference between different signals in wireless communication systems. Metasurfaces can also be used to achieve beamsteering, which allows the direction of the antenna beam to be controlled without physically moving the antenna. This feature can significantly increase the capacity of wireless communication systems by enabling multiple users to share the same frequency band. Furthermore, metasurfaces can be used to achieve signal-wave amplification, intelligent signal management and power handling, frequency conversion, analogue and digital signal modulation, signal processing and direction of arrival estimation. These features enable the development of highly sophisticated wireless communication systems that can meet the increasing demand for high-speed data transfer, low-latency communications and intelligent communication networks.

## FUNDAMENTAL BEAM MANIPULATION WITH HUYGENS’ METASURFACES

Among the first electromagnetic problems that challenged the metasurface community were those of anomalous refraction or reflection. The generalized law of refraction provided a way to tilt wavefronts with the use of metasurfaces by imparting a linear phase profile across the boundary [32]. Later, the metasurface boundary was modeled as a collection of co-located orthogonal electric and magnetic dipole moments, forming Huygens’ sources that radiate secondary fields [33,34]. In a passive implementation of these Huygens’ metasurfaces (HMSs), the electric and magnetic dipole moments are induced by the incident fields through appropriately designed scatterers, often referred to as unit cells. Regarding refraction, it was proven that omega-bianisotropy is necessary to completely eliminate reflections and anomalously refract the beam with perfect efficiency [35]. This led to the experimental demonstration of perfect reflectionless refraction with omega-bianisotropic HMSs [36,37].

Perfect anomalous reflection turned out to be fundamentally more difficult to realize, as an inspection of the power density across the metasurface shows that active and lossy regions are locally required. However, this physical requirement can be alleviated if auxiliary surface waves are introduced [38]. Alternatively, sparse periodic metasurfaces that exhibit nonlocal interactions have been utilized to achieve perfect reflection with high efficiency [39]. These implementations, which do not require a highly-subwavelength discretization, are often referred to as metagratings, and they can be designed by directly optimizing one metasurface period to maximize the coupling to desired Floquet-Bloch modes [40,41]. It is worth mentioning that static anomalous reflectors can be utilized in future smart wireless environments, when they are combined with algorithms to enhance the quality of service in areas that are not sufficiently covered by the base station [42].

## STATIC ANTENNA BEAMFORMING

One of the most promising applications of metasurfaces is to realize beams with desired characteristics without the size, complexity and power losses of conventional antenna arrays. Both microstrip-based and waveguide-based feeding networks that are commonly used in antenna arrays can introduce significant losses (e.g. at the level of 2.5–3 dB for a 16 × 16 elements array), which increase further with the larger array sizes or when amplitude tapering is required [43,44]. In addition, adding integrated electronically controlled phase shifters to the feeding network for the realization of phased arrays also contributes to losses, which reach 6 dB or more at millimeter-wave frequencies [45,46]. In contrast, passive Huygens’ metasurfaces can directly synthesize aperture fields with a very simple source element illuminating the metasurface, essentially eliminating the need of a feeding network [47]. Moreover, reconfigurability can be introduced to metasurfaces with the use of tunable elements exhibiting fewer losses (e.g. varactors, PIN diodes), as will be discussed in the next sections.

While a passive omega-bianisotropic metasurface needs to adhere to the local power conservation condition, auxiliary surface waves have emerged as a way to circumvent this constraint and achieve full control of the amplitude and phase of the synthesized aperture fields [38]. Combined with optimization techniques, this allows one to realize far-field patterns with the metasurface operating in reflection or transmission. Initially, the optimization approaches were focused on finding the auxiliary surface-wave spectrum, so that the local power conservation condition is restored locally across the entire metasurface [48–50]. Then, the metasurface parameters were analytically obtained and realized with subwavelength single-layer (for reflective metasurfaces) or multi-layer (for transmissive metasurfaces) unit cells. With this approach, the spectrum of the surface waves can be explicitly controlled, as it is introduced directly into the optimization scheme. However, modelling the metasurface as an infinitesimally thin boundary neglects the coupling effects between adjacent cells. This can be detrimental to the overall performance of the metasurface, especially when surface waves are involved, as these lead to fast-varying metasurface parameters. To alleviate this issue, copper vias can be used as baffles between the cells, restoring the transverse electromagnetic propagation within the cell but adding to the fabrication complexity [51].

With the advent of metagratings for periodic structures, the design of aperiodic metasurfaces has also been revisited, so that mutual coupling between cells is taken into account. Specifically, a finite aperiodic metasurface can be analyzed based on integral equations solved with the method of moments [52,53]. In this way, the actual topology of the metasurface (finite thickness, dielectric layers, etc.) and all coupling effects are considered and the only homogenized element is a single-layer scatterer represented with a homogenized impedance. In this case, the optimization variables are the impedance values of the scatterers and a cost function is defined directly with respect to the desired far-field pattern. Nevertheless, surface waves are still excited as the fundamental underlying physical mechanism to achieve amplitude tapering.

**Figure 2.**
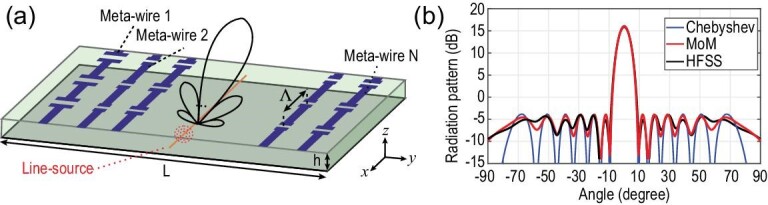
(a) Sketch of an impedance metasurface with an embedded current line source. (b) Realized Chebyshev radiation pattern with loaded meta-wires (black curve) and the homogenized MoM model used for the optimization (red curve). Reproduced from ref. [53].

Based on this approach, designs operating at 10 GHz with an embedded source were presented in [53]. The topology is sketched in Fig. 2(a), with the metasurface consisting of *N* = 42 meta-wires spanning a total width of *L* = 7λ ≈ 21 cm along the *x* axis and assumed to be infinite along the *y* axis with a periodic capacitive loading every Λ = λ/8. The wires are etched on top of a grounded dielectric slab, while a current line source is placed in the middle of the dielectric layer. By optimizing the impedance loadings of each meta-wire, the obtained far field from the method of moments (MoM) analysis is matched to a desired Chebyshev pattern with −20-dB sidelobe level. Then, the abstract impedance loadings are replaced with printed capacitors of appropriate width and full-wave simulations are performed for verification. As shown in Fig. 2(b), the results with patterned cells match quite well both the MoM predictions, but also the desired Chebyshev pattern. The copper and dielectric losses of the structure are estimated to be only $7\%$ and the 3-dB bandwidth is $6\%$ of the center frequency. Typically, the limited bandwidth is related with the auxiliary surface waves that are excited along the surface and with the frequency dispersion of the meta-wires. Regarding the surface waves, constraints were introduced in [53] to limit the relevant strength of the evanescent spectrum and, in general, obtain more broadband solutions. While these constraints can be useful, they limit the solution space and, gradually, the obtained converged solutions do not much that closely with the desired radiation pattern. Another idea is to actually predict the bandwidth (or at least the performance in some adjacent frequencies) during optimization, while considering the frequency dispersion of the meta-wires through an extracted model from simulation. This would generally make the optimization process more complicated and time consuming, but it could be investigated as a way to obtain designs with enhanced bandwidth.

Another well-established technique for designing static metasurfaces for realizing antenna beams without feeding networks is the holographic approach [54]. In this case, a feed point excites a surface-wave mode that is supported on an impedance layer above a grounded substrate. A spatial modulation can then be determined for the impedance values, so that the surface-wave mode is gradually converted to a leaky-wave mode towards the desired propagating direction [55]. Controlling locally the modulation depth allows some control over the amplitude of the aperture fields, while a tensor formulation allows for polarization control [56]. Based on the holographic metasurface antennas, various realizations have been demonstrated, including circularly polarized beams with broadside and isoflux radiation patterns [55,56]. Proper design of the feed and the leakage constant, combined with low loss dielectric substrates can result in overall aperture efficiencies of 75% even for electrically large apertures (e.g. radius of 20 wavelengths) [57].

## DIELECTRIC MODULATED METASURFACES

Similar functionalities can be demonstrated with a metasurface consisting of a dielectric substrate of varying thickness above a ground plane. Avoiding copper-based cells may be beneficial at higher frequencies, where Ohmic losses and fabrication complexity become constraining factors. On the other hand, dielectric slabs with modulated thickness can be easily fabricated with little cost through three-dimensional (3D) printing. Early efforts to realize beams with a thickness-modulated dielectric metasurface were based on the holographic approach, where a sinusoidal modulation on the effective impedance of the dielectric (or its thickness) leads to gradually converting the propagating mode to leaky-wave radiation [58]. Another approach is to optimize the necessary surface-wave spectrum for a particular wave transformation, as done with copper-based metasurfaces, and then realize locally the effective impedance with a dielectric substrate of varying thickness [59]. Finally, integral equations combined with the method of moments can be utilized to directly optimize the thickness of different segments of the dielectric in order to realize desired far-field patterns [60].

**Figure 3.**
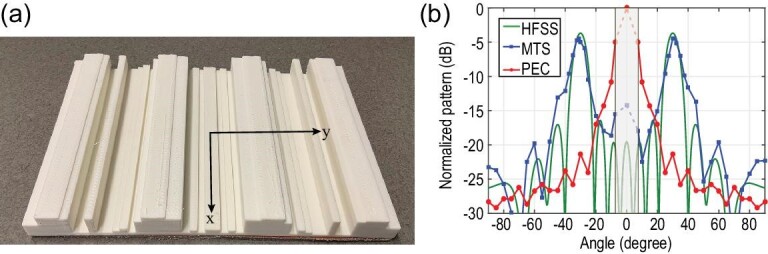
(a) Fabricated prototype for beam splitting at 10 GHz. (b) Measured results (blue curve) for the metasurface (MTS) compared with the scattering of a perfect electric conductor (PEC) for reference (red curve) and with the simulated pattern (green curve). Beam splitting is achieved with high-efficiency and low sidelobe levels. Reproduced from ref. [60].

The last method can be utilized both with an embedded source forming a dielectric based aperture antenna, and with external illumination as a reflective surface with a desired reflected radiation pattern. The latter case was experimentally demonstrated in [60], where a beam-splitting dielectric metasurface was designed. The metasurface is 7λ at the operating frequency of 10 GHz and it is divided into 48 pieces of varying thicknesses. By optimizing the thickness of each segment, we can realize two reflected beams at ±30° with high efficiency. The 3D-printed prototype, shown in Fig. 3(a), consists of the modulated dielectric (printed with acrylonitrile butadiene styrene) and a copper base layer. Illuminating the metasurface with a normally incident beam from a horn antenna, the reflected field values are recorded. As observed in Fig. 3(b), the reflected pattern matches well the theoretical one, as two beams are realized at the expected angles. Moreover, the measured peaks are only 0.8 dB lower, when compared to the lossless theoretical directivity pattern, suggesting low power losses for the printed dielectric layer. Lastly, both peaks in measurement remain within 1 dB of their maximum value for the range 9.05 to 10.5 GHz. As expected, the dielectric design exhibits, in general, a more broadband response compared to copper-based implementations presented in the rest of the sections.

## MULTI-INPUT/MULTI-OUTPUT BEAM MANIPULATION

It can be advantageous for a metasurface to handle multiple inputs and generate multiple output beams. For applications in wireless communications, this translates to multiple communication links being established with the use of a single metasurface aperture. The concept has been studied in the context of metasurface holographic antennas where multiple feeds realize multiple beams in different directions [61]. Specifically, a ‘multiplexing’ technique was presented where the overall homogenized impedance is set to be the summation of the individual impedance profiles as emerging from the holographic approach for each input channel. While this method offered higher aperture efficiency compared to simply partitioning the metasurface aperture, a synthesis of the metasurface based on direct optimization of the impedance layer for all inputs could potentially offer even higher aperture efficiencies. To this purpose, the integral equations and method of moments framework presented in the previous sections is deemed suitable, as at any iteration of the optimization the output for all inputs can be fully predicted. Thus, by defining a cost function that takes into account all the desired output beams, it is possible to obtain optimization solutions beyond the heuristic ‘multiplexing’ approaches in the literature. Such metasurface designs could refer to multiple frequencies producing prescribed radiation patterns, multiple embedded sources realizing different beams or multiple incident plane waves reflecting at multiple prescribed directions [62,63].

Here we report on recent results for multi-input multi-output metasurfaces using the method of moments optimization method [63]. Specifically, a metasurface with 42 meta-wires across a total length of 8λ is designed at 10 GHz to anomalously reflect two incident beams from θ_in, 1_ = −20° and θ_in, 2_ = +20° to θ_out, 1_ = 0° and θ_out, 2_ = 40°, respectively. The homogenized impedance loadings that are determined through optimization are implemented with printed capacitive loadings every Λ = λ/6. A Rogers RO3003 substrate (ϵ_*r*_ = 3, tan(δ) = 0.001) with thickness *h* = 1.52 mm is picked as the grounded dielectric. Full-wave simulation results of the optimized solution showed highly directive reflected beams for both cases (less than 0.5-dB difference from a uniform-amplitude aperture radiating towards the desired direction), while maintaining losses below $10 \%$ of the incident power. Simultaneously, the bandwidth for a 3-dB drop in the gain towards the desired output direction was estimated at $8.4 \%$ and $6.8 \%$ (of the center frequency) for the beams at θ_out, 1_ = 0° and θ_out, 2_ = 40°, respectively.

**Figure 4.**
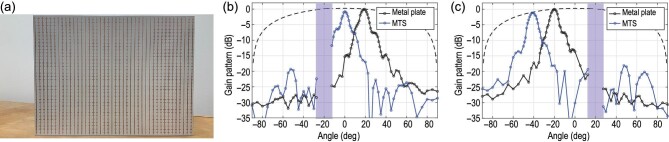
(a) Fabricated prototype for anomalous reflection of two incident plane waves at 9.7 GHz. (b) Measured gain pattern of the reflected beam for incident illumination at θ_in, 1_ = −20°. (c) Measured gain pattern of the reflected beam for incident illumination at θ_in, 1_ = +20°. The shaded areas represent zones for each case that measurements cannot be performed due to limitations of the measurement setup.

The fabricated prototype is depicted in Fig. 4(a), and it is measured in a bi-static measurement setup comprising a transmitting horn antenna at a fixed location and a rotating receiving horn antenna. A slight shift in the frequency of operation was observed in the measurements; thus, the reported results are at 9.7 GHz, which corresponds to a shift of the substrate’s dielectric constant to ϵ_*r*_ = 3.25 according to simulations. However, the beams are indeed reflected in the desired output angles, i.e. 0° and −40° for the two designed cases. To evaluate the metasurface patterns, the reflected patterns of a metallic plate of the same size are also measured, when illuminated from θ_in, 1_ = −20° and θ_in, 2_ = +20°. The maximum of the reflected patterns from the metal plate, which occurs in the specular direction, is used to scale the graphs in Fig. 4(b and c), while the expected steering loss (due to the effective reduction of the aperture size at oblique angles) is also included as a dotted line. As observed, the beam at 0° for the first case has a degradation of 1.1 dB compared to the expected value lying on the dotted line, while the beam at −40° for the second case matches closer with only a 0.2-dB degradation. Moreover, the specular reflection is highly suppressed (>17-dB reduction) for both incident angles.

## RECONFIGURABLE INTELLIGENT SURFACES

Communication systems need to continuously evolve to meet the growing requirements regarding the total amount of connected devices and the provided quality of service. Sixth generation (6G) communications and beyond are expected to cope with these demands not only by more sophisticated hardware and software at the transmitting and receiving end, but also by making full use of the communication channel. To this end, reconfigurable intelligent surfaces (RISs) that are able to redirect beams have been studied both from a communication and a physical design standpoint [64]. RISs can reflect the incident beams to off-specular directions, providing significant enhancement for the received power when the line of sight is blocked by objects [65]. Moreover, as the name suggests, these surfaces are dynamically controlled through electronically tunable elements (e.g. PIN diodes, varactors) in order to modify their properties and functionality. In the following, we report on recent advancements regarding the design and implementation of RISs from an electromagnetic perspective.

Conventionally, RISs have been implemented by means of reconfigurable reflectarrays, where each tunable element (unit cell) provides locally a tunable phase for the reflected field [66,67]. By adjusting the phase step between consecutive cells, it is possible to have constructive interference at the desired off-specular direction. The unit cells can provide a discrete number of phase levels through one or more PIN diodes, or a continuous range with the use of varactors [68–72]. Losses are generally lower for RISs compared to phased arrays that include phase shifters, making them attractive for beamforming applications. For instance, in [71] a $59\%$ reduction in power consumption was reported compared to a conventional phased array of similar gain. While beamsteering of the reflected beam is the most-commonly demonstrated functionality of RISs, reconfigurable polarization conversion can also be achieved by varactor-loaded cells [73]. In addition, metasurfaces for simultaneous tunable transmission and reflection, known as intelligent omni-surfaces, have been designed for cases that full 360° coverage is required [74]. Lastly, RISs can be combined with amplifiers in a multi-layer implementation to enhance the incident signal in addition to performing any tunable beam manipulation [25,26].

In the vast majority of RIS designs, only the phase of the reflected fields is modified, as the amplitude profile solely depends on the incident illumination. Moreover, the inter-element coupling effects are often neglected, since the reflectarray element is designed as if it is placed in an infinitely large uniform array. On the other hand, it has been shown that auxiliary surface waves can be introduced in the design of metasurfaces to taper both the amplitude and the phase of the scattered fields. In this case, the cells are usually spaced closer than λ/2 (as in traditional reflectarrays) to support the necessary spectrum of evanescent waves [75]. These surface waves can be introduced through optimization to facilitate the desired field transformation, similar to the static designs presented previously. In the rest of this section, we discuss the design of an RIS based on an integral equation formulation with the aim of independent amplitude and phase control for the reflected fields.

The designed RIS operates at 5 GHz and it has a width of *L_y_* = 7λ ≈ 42 cm along the *y* axis, while it is assumed to be infinitely long along the *x* axis, similar to the geometry of Fig. 2. The impedance layer is etched on a Rogers RO5880 substrate with dielectric constant ϵ_*r*_ = 2.2, loss tangent tan(δ) = 0.001 and thickness *h* = 3.125 mm. It consists of 31 meta-wires that are periodically loaded every Λ = λ/4 with unit cells incorporating varactors as a tuning mechanism. The homogenized impedance value of each meta-wire is optimized based on the integral equation framework solved with the method of moments that was presented in [53]. Then, the impedance values are matched to the corresponding DC bias voltages for the varactors at each meta-wire.

**Figure 5.**
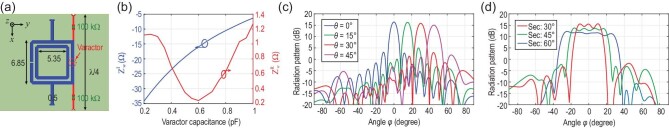
(a) Geometry of the reconfigurable varactor-loaded cell at 5 GHz. (b) Extracted loading impedance $Z_{\scriptsize\textit{w}}=Z_{\scriptsize\textit{w}}^{\scriptsize\textit{R}}+jZ_{\scriptsize\textit{w}}^{\scriptsize\textit{I}}$ as a function of the capacitance of the varactor. (c) Beam steering at 0° to 45° with maximum directivity (negative angles are also possible by symmetrically mirroring the bias voltages). (d) Sector patterns of varying beamwidth.

The unit cell consists of two coupled square loops, where the outer loop is loaded with a MACOM MAVR-000120 varactor, as shown in Fig. 5(a). The varactor is modelled in electromagnetic simulations as a lumped RC element with capacitance and resistance as extracted from Keysight Advanced Design System (ADS) based on its spice model. Bias lines are included in the unit cell loaded with *R* = 100-KΩ resistors to dissipate any induced radio-frequency (RF) power to them. These bias lines are connected to the middle layer (ground plane) and the back bias lines through vias, such that a reverse bias is always applied to all the varactors on each meta-wire. The unit cell topology provides a response equivalent of two parallel LC networks, and has been utilized in the past due to its wide and smooth variation of impedance values [76]. Specifically, by characterizing the equivalent loading impedance of the cell we are able to optimize the geometric features so that a sufficient range is obtained within the varactor’s capacitance range. The characterization happens in an aperiodic environment, where a single meta-wire is simulated in Ansys High-Frequency Simulation Software (HFSS) and the near-field values are compared with the method of moments solution of the homogenized model. This establishes a correspondence between the varactor’s capacitance (or bias voltage) and the complex $Z_w=Z_w^R+jZ_w^I$ loading impedance values, as plotted in Fig. 5(b). We note that, for this tunable unit cell, it is quite important to characterize the cell, including the real part of the loading impedance, as it is related to the losses induced by each cell. While the imaginary parts still constitute the optimization variables in the method of moments framework, the real part is supplemented to accurately predict the power losses. As a result, by introducing a power efficiency constraint, we are able to converge to solutions that exhibit less power losses.

Two types of examples are investigated with the designed RIS. In the first one, the RIS is illuminated by a normally incident plane wave and the ability to steer the beam is explored. By optimizing for maximum directivity at the reflected angles θ_*r*_ = 0°, 15°, 30° and 45°, we obtain the necessary sets of impedance loadings for each meta-wire. It is noted that the imaginary part $Z_w^I$ is constrained in the range $-30 \le Z_w^I \le -8$ in order to be realizable with our designed cell. Additionally, power efficiency (including the varactors’ losses) is constrained at $85 \%, 76\%, 75\%$ and $72\%$ for the four output angles. After obtaining the impedance values and the corresponding bias voltages, full-wave simulations are performed in HFSS based on the actual patterned structure to assess its performance. From Fig. 5(c), it is verified that the beam indeed steers at the desired output angles with high directivity. To assess the efficiency of the RIS, we define the illumination efficiency η_il_ as the ratio between the simulated directivity from the RIS and that obtained from an ideal uniform-amplitude aperture of the same size that radiates towards the desired direction. For all cases, the obtained directivity differs by less than 0.2 dB from the ideal directivity of a uniform aperture, resulting in illumination efficiencies $\eta _\mathrm{il}>97 \%$. In addition, the calculated power efficiency is within $2 \%$ of the imposed constraints, with the most lossy case of a reflected angle θ_*r*_ = 45° exhibiting $71\%$ power efficiency. The bandwidth for a 3-dB drop in the simulated directivity is 3.4%–3.5% of the center frequency (for all the cases of anomalous reflection from 15° to 45°), limited primarily by the frequency dispersion of the cells comprising the RIS.

In the second example, the possibility of amplitude tapering is demonstrated by realizing sector patterns of varying beamwidth. The RIS is illuminated by an incident Gaussian beam 30° off-broadside with a waist of 3λ, while the output beam is centered at broadside. Sector patterns of 30°, 45° and 60° are set as the desired far field with a power efficiency of at least $65 \%$, including all losses present in the structure. The radiation patterns from full-wave simulations with the patterned cell and the optimized bias voltages are given in Fig. 5(d). For all cases, the ripple in the passband remains below 1.7 dB, the sidelobe level is at least −13 dB and the power constraint is satisfied. We note that the losses are slightly higher compared to the beamsteering example, due to the stronger surface-wave spectrum in the vicinity of the metasurface that is necessary to achieve the amplitude tapering.

## BEAMSTEERING AND THE SIGNAL BOOSTING NONRECIPROCAL REFLECTIVE METASURFACE

**Figure 6.**
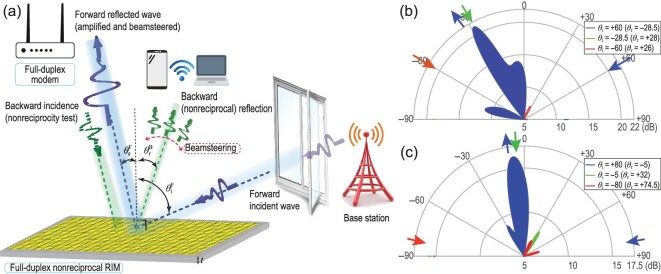
Reflective dynamic metasurface for nonreciprocal beamsteering and amplification. (a) Functionality of the metasurface. (b) Experimental results for wave incidence at 60°. Reproduced from ref. [13]. (c) Experimental results for wave incidence at 80°. Reproduced from ref. [13].

In Fig. 6 we demonstrate the experimental implementation of a reflective dynamic metasurface for nonreciprocal beamsteering and amplification [13]. This reflective metasurface is composed of an array of phase-gradient cascaded radiator-amplifier-phaser unit cells. The unit cell of this reflective metasurface is composed of a patch radiator, a unilateral circuit and a reciprocal phase shifter. An electromagnetic wave impinging on top of the reflective metasurface from the right side (forward incident wave) under the angle of incidence ${\theta _{i}}^{F}$ is reflected and steered towards a desired direction in space under the angle of reflection ${\theta _{r}}^{F}$, while possessing the same frequency as the frequency of the impinged wave. The backward incidence assumes the time-reversed version of the forward incidence, where an electromagnetic wave impinges on top of the reflective metasurface from the left side under the angle of incidence ${\theta _{r}}^{F}$. Then, it is amplified by the reflective metasurface and is being reflected towards a desired direction in space under the angle of reflection ${\theta _{r}}^{{B}} \ne {\theta _{{i}}}^{F}$. Such a nonreciprocal reflection is evidently different than the reflection by a reciprocal surface where ${\theta _{{r}}}^{{B}} = {\theta _{{i}}^{{F}}}$. The metasurface is composed of a dielectric layer sandwiched between two conductor layers, where the conductor layers are composed of a plurality of unit cells embedded therein. The operation of the unilateral transistors and unit cells is controlled via a DC biasing signal, thereby arbitrary phase shift and amplitude profiles can be created along the metasurface.

Such reflective metasurfaces can be mounted on a roof, wall or on a smart device in a seamless manner. They may be further developed for massive MIMO beamforming, where multiple beams and multiple frequencies are supported, and no cumbersome RF feed lines and matching circuits are required. The reflective metasurface functionality is fully controlled through DC biasing of the transistors, variable phase shifters and tunable patch radiators. Such a highly directive and reflective nonreciprocal beamsteering represents a promising feature to be utilized for low-cost high capability and programmable wireless beamsteering applications. Such a reflective metasurface may act as the core of an intelligent connectivity solution for signal boosting in cellular, WiFi and satellite receivers and internet-of-things sensors, introducing high speed scanning between users, multiple access and signal coding.

## NONRECIPROCAL-BEAMSTEERING TRANSMISSIVE METASURFACE

**Figure 7.**
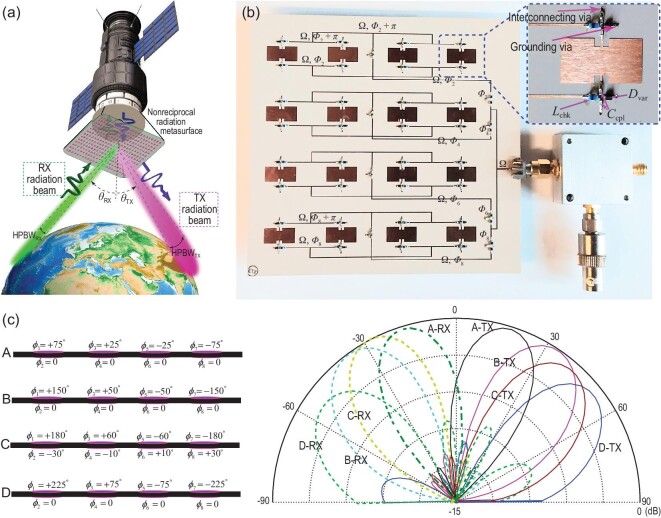
Nonreciprocal-beam transmissive phase-engineered temporal metasurface. (a) Application to an efficient point-to-point satellite communications. Reproduced from ref. [12]. (b) A photo of the fabricated prototype. Reproduced from ref. [12]. (c) Experimental results for nonreciprocal beamsteering (right-hand side) corresponding to different sets of gradient nonreciprocal phase shifts (left-hand side). Reproduced from ref. [12].

In Fig. 7 we show an experimental demonstration of the nonreciprocal-beam transmissive temporal metasurface for efficient point-to-point wireless communications [12]. Such a transmissive metasurface provides different radiation beams for transmission and reception, i.e. different radiation angles and half-power beamwidths, as *F*_TX_(θ) ≠ *F*_RX_(θ), where *F*_TX_(θ) = *E*_θ, TX_/*E*_θ, TX_(max) is the transmission radiation pattern, and *F*_RX_(θ) = *E*_θ, RX_/*E*_θ, RX_(max) is the reception radiation pattern. The realization of such a nonreciprocal transmissive metasurface is accomplished through an array of time-modulated phase-gradient unit cells.

Such a functionality is achieved by leveraging the unique properties of asymmetric frequency-phase transitions in time-modulated unit cells, where all the unwanted time harmonics are suppressed in an elegant manner. In addition, there is no inherent limit to the bandwidth enhancement of the proposed transmissive metasurface. The time modulation is practically achieved through biasing of varactor diodes by a DC signal and an RF time-varying signal. The DC and RF signals are then used for controlling the functionality of the metasurface. For instance, the transmission and reception beams of the metasurface can be steered by phase shifting the RF modulation signal, as well as altering and the magnitudes of the transmission and reception beams can be tuned through the amplitude of the RF modulation signal bias.

## FREQUENCY-BEAMSTEERING TRANSMISSIVE METASURFACE

**Figure 8.**
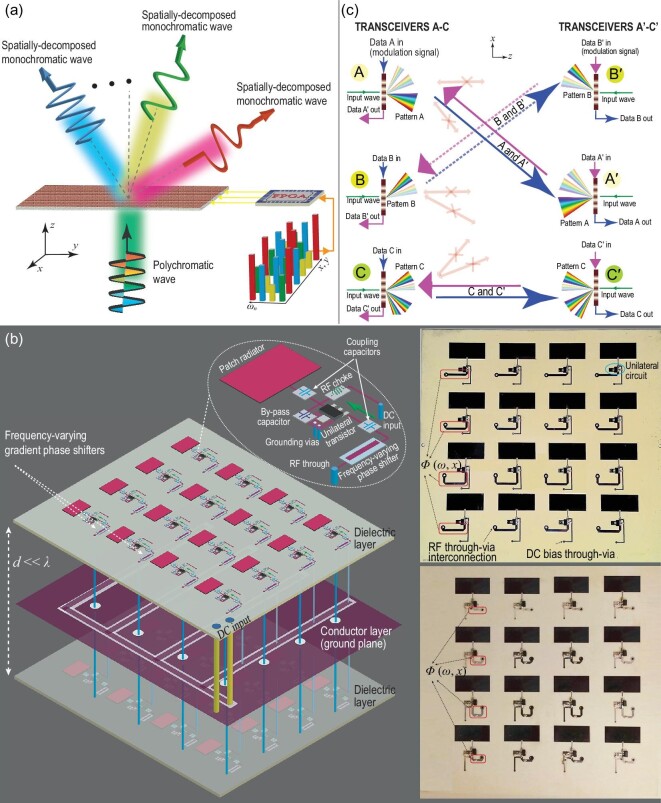
Polychromatic metasurfaces. (a) Frequency-beamsteering (prism functionality) transmissive metasurface. Reproduced from ref. [24]. (b) Fabricated prototype. Reproduced from ref. [24]. (c) Space-time diffraction-code multiple access system based on real-time pattern coding of space-time diffraction transmissive metasurfaces. Reproduced from ref. [16].

Figure 8(a) sketches the functionality of a frequency-beamsteering transmissive metasurface. Such a transmissive metasurface provides a frequency-dependent spatially variant phase shift and a frequency-dependent spatially variant signal gain [24]. We assume that a polychromatic electromagnetic wave ${\bf \psi }_{\rm in}$ that comprises various frequency components, ω_1_, ω_2_, …, ω_N_, passes through the transmissive metasurface, where the transmissive metasurface introduces frequency-dependent spatially variant phase shifts. Hence, the frequency components of the incident polychromatic wave acquire different phase shifts at each point in the *x*–*y* plane, i.e. φ_*n*_(*x, y*). The phase and magnitude profiles of the transmissive metasurface, i.e. φ(ω, *x, y*) and *T*(ω, *x, y*), are engineered in a way that each transmitted frequency component ${\bf \psi }_{\rm out}^{f_n}$ is transmitted under a desired transmission angle $\theta _{\rm t}^{f_n}$ with a specified signal gain of $|{\bf \psi }_{{\rm out},n}|$.

Figure 8(b) shows the architecture and a photo of the fabricated frequency-beamsteering transmissive metasurface. The frequency-beamsteering transmissive metasurface is composed of an array of active frequency-dependent spatially variant unit cells introducing the required phase shift and magnitude for efficient beamsteering and signal amplification. The experimental results show favourable features such as prism-like spatial decomposition of frequency components, possible isolation between the forward and backward transmissions, and a desirable signal gain. Furthermore, the frequency-beamsteering transmissive metasurface is fairly broadband and its bandwidth can be further increased by standard band-broadening methods.

The frequency-beamsteering transmissive metasurface introduces a specified beam direction for each frequency component, as well as amplification. However, the directions of the beams at different frequencies can be easily controlled via the DC bias of the transistors, as well as the phase shift provided by the gradient phase shifters. Therefore, a diverse range of transmission angles and amplifications is achievable by adjusting the DC voltage of the transistors and variable gradient phase shifters. Additionally, such a metasurface is immune to undesired and problematic time harmonics that are produced in time-modulated metasurfaces. As a result, such a transistor-based frequency-beamsteering transmissive metasurface offers a unique and efficient response to be employed for several practical applications. Our lab experiments show that such a metasurface can boost data throughput by more than $200\%$.

## DIFFRACTION-CODE MULTIPLE ACCESS SYSTEM

Figure 8 sketches a space-time diffraction-code multiple access system that is based on real-time diffraction pattern generation from space-time diffraction transmissive metasurfaces [16]. Such a coding transmissive-metasurface-assisted system offers unique properties that can be utilized for advanced and versatile real-time signal coding and beamsteering. In the particular example in Fig. 8, three pairs of transceivers are considered, but in practice one may consider more pairs of transceivers. Then, only the transceiver pairs sharing identical space-time diffraction patterns are communicating effectively. Each diffraction pattern is attributed to the properties of the space-time-modulated transmissive metasurface, that is, the input frequency, where the input data (message) play the role of the modulation signal. For a specified input modulation data signal, a unique diffraction pattern is achieved. Here, the transceiver pairs that are allowed to communicate are 1 and 1′, 2 and 2′, and 3 and 3′, whereas transceivers 2′ and 3′ (2 and 3) cannot retrieve the data sent by transceiver 1 (1′), and transceivers 1′ and 3′ (1 and 3) cannot retrieve the data sent by transceiver 2 (2′). Distinct diffraction patterns can be created by certain space-time modulation parameters, e.g. the modulation amplitude, modulation frequency and transmissive metasurface thickness. In addition, the radiation pattern provided by a space-time transmissive metasurface is very diverse and is sensitive to the space-time modulation parameters. Hence, an optimal isolation between the transceivers can be achieved by proper engineering of the space-time diffraction patterns.

## FREQUENCY CONVERTER METASURFACE

**Figure 9.**
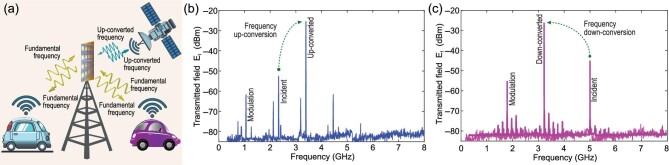
Pure frequency converter temporal transmissive metasurface. (a) Application to satellite and urban wireless communications. Experimental results for (b) up-conversion and (c) down conversion. Reproduced from ref. [15].

Frequency conversion is a crucial task in wireless communications for adaptation of the transmitted signal depending on the communication channel characteristics for lower path loss. In Fig. 9 we show functionality and experimental results of the pure frequency converter transmissive metasurface to satellite and cellular communications [15]. Here, the metasurface is placed on top of a base station to create an efficient connection between the cellular base stations and mobile phones and satellites. In particular, consider the cellular phones operating in the Broadband Personal Communications Service (PCS Band), i.e. 1850–1910/1930–1990 MHz, and the satellite network operating in the C band, i.e. 3700 and 7025 MHz, which is a well-known frequency band for satellite communications. Hence, the transmissive metasurface makes an efficient connection between the cellular and satellite networks. To improve the functionality of the temporal transmissive metasurface in this application, appropriate beamsteering mechanisms [12,13,24] can be added to the frequency conversion functionality of the transmissive metasurface.

## DISCUSSION

Frequency conversion and time harmonics of temporal metasurfaces may be employed in different schemes and for various applications, such as for pure frequency conversion [27] and antenna-mixer-amplifier multifunctional operation [20]. Furthermore, a space-time-coding digital metasurface can be created based on temporal modulation of the reflection coefficient [30]. Such a metasurface generates an optimal harmonic scattered-power distribution via a set of coding sequences that are being switched cyclically in a predesigned time period. In addition, frequency conversion of time metasurfaces can lead to a microwave nonreciprocal metasurface operating based on one-way surface-wave generation [23]. Such a metasurface reflects the forward incident beam into far-field radiation while it translates the backward incident wave into near-field surface waves. A serrodyne frequency translator employs linear sawtooth modulation of transit time to shift the frequency of a signal [28,29].

The transmissive efficiency of such a temporal frequency converter metasurface depends on different parameters of the unit cells of the metasurfaces, including the incident frequency, patch radiators, modulation frequency, modulation amplitude and Ohmic resistance of varactor diodes. With proper design of the dispersion diagram of the structure that follows the modulation parameters, a transmissive efficiency of higher than $90\%$ can be achieved where part of the modulation signal power is coupled to the converted output signal.

In another study, an accurate 2D direction-of-arrival (DOA) estimation is achieved by an active metasurface undergoing nonuniform time modulation, where DOA estimation is achieved through information of multisource directions by a single-channel system [31]. In wireless communications, DOA estimation is an important technique for determining the location of a transmitter or receiver in a wireless network, and is widely used in areas such as radar systems, wireless localization and satellite communications. The relationship between DOA estimation and temporal metasurfaces lies in the fact that metasurfaces can be used to enhance the performance of DOA estimation algorithms. For example, by using a temporal metasurface to shape the radiation pattern of an antenna array, it is possible to improve the resolution and accuracy of DOA estimation. Similarly, by using a metasurface to manipulate the phase and polarization of incident waves, it is possible to enhance the robustness of DOA estimation algorithms to multipath and interference. Overall, the relationship between DOA estimation and metasurfaces in wireless communications is a synergistic one, with each field complementing and enhancing the other.

The proposed nonreciprocal and time-varying metasurfaces hold significant potential for bandwidth enhancement through strategic engineering techniques that target the patch resonators. Such techniques offer virtually limitless possibilities for enhancing the frequency bandwidth, as there is no inherent limit to the bandwidth. This is further supported by recent research studies such as [77,78], which demonstrate the effectiveness of engineering approaches in achieving broadband responses in patch elements. The controlled supply of DC bias to the transistors, time-modulated varactors and gradient phase shifters enables efficient and targeted control over the broadband response of the proposed unit cells, making them highly desirable for a range of applications.

In varactor-based time-varying metasurfaces, changing the modulation frequency affects the electromagnetic response of the metasurface and, therefore, its ability to manipulate the incident waves. Specifically, the modulation frequency determines the rate at which the varactor diodes in the metasurface switch, which in turn controls the effective electromagnetic properties of the metasurface, such as the phase and amplitude of the output waves. According to the Manley-Row relation [79], the power of the up- and down-converted time harmonics corresponds to the ratio of the modulation frequency and the input frequency. Hence, changing the modulation frequency directly affects the amplitude of the output time harmonic. Additionally, the relationship between the main input signal frequency and the temporal modulation frequency in time-varying metasurfaces depends on the specific application and design parameters of the metasurface. For instance, in the frequency converter metasurface [15], the unidirectional beam-splitter metasurface [80] and the coherency-based isolator [81], a high temporal modulation frequency is utilized that is of the order of the input frequency, so that the sum (or difference) of these two frequencies results in a practical frequency conversion where the resultant frequency falls in a higher (or lower) frequency band. In contrast, for the nonreciprocal-beam temporal frequency application, we considered a low temporal modulation frequency, such that all the desired and undesired time harmonics fall inside the frequency band of the patch elements and are controlled (either transmitted or suppressed) by the temporal unit cells of the metasurface.

## CONCLUSION

Metasurfaces are two-dimensional reconfigurable, passive/dynamic and energy-efficient structures that are capable of reshaping the wireless environment for an enhanced throughput. They introduce appealing physical features and functionalities such as gain, beamsteering and beamforming, spatial frequency decomposition, nonreciprocal signal transmission, frequency conversion, nonreciprocal-beam radiation and real-time pattern coding. Such features make reconfigurable transmissive metasurfaces and reflective metasurfaces promising solutions for enabling future communication-intensive applications such as smart cities. These structures can optimize the spectral efficiency for single and multiple carrier communication systems. In addition, they can provide a complex bit/power allocation on different spatial-domain subchannels for an improved bit error rate performance, and present a spatial-decomposition-based beamforming and beamsteering platform for MIMO systems. In this article, we have discussed some recent experimental results on different reconfigurable metasurfaces that can be utilized for a variety of applications in wireless communications and beyond.

While the research on metasurfaces has evolved significantly over the last two decades, challenges for their integration in wireless communications still need to be addressed. In particular, the cost of fabricating metasurface prototypes is usually high and designs are often impractical for mass low-cost production. Furthermore, there is a lack of experiments in realistic wireless environments, where dynamic phenomena such as multi-path interference play an important role. On the other hand, there is a need to further enhance the electromagnetic performance of metasurfaces on practical metrics, such as the operating bandwidth that is often limited by the resonant response of the constituent cells and the power efficiency associated with material loss or dissipation on the tunable components. Moreover, efficient algorithms need to be developed from a communication perspective so that the capabilities of multiple metasurfaces in a wireless environment are fully utilized to enhance the coverage, data rate and overall quality of service of communications. Finally, practical and aesthetic issues should be carefully considered when deploying such metasurfaces in indoor and outdoor environments.
